# Decreased piRNAs in Infertile Semen Are Related to Downregulation of Sperm MitoPLD Expression

**DOI:** 10.3389/fendo.2021.696121

**Published:** 2021-07-13

**Authors:** Yeting Hong, Yanqian Wu, Jianbin Zhang, Chong Yu, Lu Shen, Hanxiao Chen, Linjie Chen, Xue Zhou, Fang Gao

**Affiliations:** ^1^ College of Laboratory Medicine, Hangzhou Medical College, Hangzhou, China; ^2^ Zhejiang Provincial People’s Hospital, People’s Hospital of Hangzhou Medical College, Hangzhou, China; ^3^ College of Laboratory Medicine and Life Sciences, Wenzhou Medical University, Wenzhou, China; ^4^ Department of Andrology, Nanjing Drum Tower Hospital, Affiliated Hospital of Nanjing University Medical School, Nanjing, China

**Keywords:** infertility, piRNAs, exosome, sperm, MitoPLD

## Abstract

Currently, the molecular mechanisms underlining male infertility are still poorly understood. Our previous study has demonstrated that PIWI-interacting RNAs (piRNAs) are downregulated in seminal plasma of infertile patients and can serve as molecular biomarkers for male infertility. However, the source and mechanism for the dysregulation of piRNAs remain obscure. In this study, we found that exosomes are present in high concentrations in human seminal plasma and confirmed that piRNAs are predominantly present in the exosomal fraction of seminal plasma. Moreover, we showed that piRNAs were significantly decreased in exosomes of asthenozoospermia patients compared with normozoospermic men. By systematically screening piRNA profiles in sperms of normozoospermic men and asthenozoospermia patients, we found that piRNAs were parallelly reduced during infertility. At last, we investigated the expression of some proteins that are essential for piRNAs biogenesis in sperms and therefore identified a tight correlation between the levels of spermatozoa piRNA and MitoPLD protein, suggesting that the loss-of-function of MitoPLD could cause a severe defect of piRNA accumulation in sperms. In summary, this study identified a parallel reduction of piRNAs and MitoPLD protein in sperms of asthenozoospermia patients, which may provide pathophysiological clues about sperm motility.

## Introduction

Infertility is a prevalent health problem and affects nearly 15% of couples all over the world (1, 2). Male factors contribute to about 50% of childless couples. As a complex disease, male infertility is caused by a series of multifactorial genetic and environment factors, but the underlying molecular mechanisms have not yet been elucidated (3–5).

piRNAs are approximately 26-31 nucleotides in length and expressed mainly in pachytene spermatocytes and round spermatids in the testis of mammals (6–9). They are named PIWI-interacting RNAs because of their close relationship with the PIWI subfamily members. Two pathways for piRNA biogenesis have been identified: namely, primary and secondary pathways (10–12). The primary pathway is thought to produce piRNAs (primary piRNAs) from long single-strand piRNA precursors, which are derived from genomic regions called piRNA clusters. The pathway about primary piRNAs generation is not well studied, but a mitochondrial protein, MitoPLD (also known as Zucchini or PLD6), a member of the nuclease*/*phospholipase D family, has been proposed to function as an endonuclease to generate the 5’ ends of piRNAs (13–15). In the secondary pathway, piRNAs (secondary piRNAs) are produced by the ping-pong amplification cycles from the 5’ portions of RNA fragments cleaved by PIWI-piRNA complexes. The piRNAs from primary and secondary pathway guide each other’s production in the ping-pong cycle to accelerate production of piRNAs (12, 16, 17). Currently, the main proposed function of piRNAs is to protect the germline and gonadal somatic cells and to avoid transposable elements related harmful expression and thus maintains the genomic integrity of germ cells. Increasing evidence has also shown that piRNAs may be involved in post-transcriptional regulation of protein-coding genes (18–20). Because of the diverse and pivotal roles of piRNAs in the male reproductive system, the dysregulation and dysfunction of piRNA often cause male infertility.

Since piRNAs are specifically expressed in germ-cell and essential for spermatogenesis, it is not surprising to find that the levels of spermatozoa piRNAs are directly correlated to semen quality and male fertility. We have previously shown that the concentration of seminal plasma piRNAs were significantly decreased in infertile patients compared with the normozoospermic men. Several specific piRNAs in seminal plasma were even identified as molecular biomarkers for male infertility (3). However, the source of seminal plasma piRNAs remains elusive, and the cause of massive reduction of piRNAs in seminal plasma of infertile patients has not been definitively identified. Extracellular RNA profile in human semen was comprehensively characterized in a recent study and a great number of small RNAs were found within seminal exosomes (21). Thus, it is rational to speculate that piRNAs in seminal plasma are mostly derived from the secretion of exosomes from the male germ cells. In this study, we validated that a majority of piRNAs were present within the exosomal fraction of seminal plasma. Moreover, we showed the piRNAs’ types and levels were significantly decreased in the sperms of asthenozoospermia patients compared with those in normozoospermic men. Finally, we identified a tight correlation between the levels of spermatozoa piRNA and MitoPLD protein, suggesting that the loss-of-function of MitoPLD could cause severe defects in the piRNA accumulation in sperms.

## Materials and Methods

### Semen Samples

Semen samples were provided from Nanjing Drum Tower Hospital, and all protocols in this study were approved by the Medical Ethics Committee of Hangzhou medical college and Nanjing Drum Tower Hospital. Informed consents were signed by both normozoospermic men volunteers and asthenozoospermia patients before sample collection for the further study. The study recruited 42 asthenozoospermia patients with infertility more than 2 years and 41 normozoospermic men volunteers who conceived naturally within 1-2 years. The demographic characteristics of all tested persons were listed in **Table 1**. The volunteers in this study did not receive any treatment, semen samples were analyzed in the Reproductive Laboratory of Nanjing Drum Tower Hospital.

**Table 1:** Demographic and clinical features of the Asthenospermia patients and normozoospermic men^a^.

Variables (sequencing set)	Normozoospermic men (n=10)	Asthenospermia patients (n=10)	*P*-Value^b^
Average age,years	28.4 (2.41)	29.9 (3.57)	0.2789
Sexual abstinence time,days	4.1 (0.74)	3.9 (0.88)	0.5554
Semen parameters			
pH	7.53 (0.28)	7.44 (0.16)	0.2869
Total volume,mL	4.62 (6.71)	4.22 (0.77)	0.2003
Sperm parameters			
Sperm density,×10^6^/mL	47.84 (14.23)	43.37 (10.34)	0.4916
Sperm viability,%	70.39 (4.94)	12.75 (7.03)	3.18×10^-9^
Progressive motility (PR)^e^	51.9 (7.09)	5.99 (4.46)	6.36×10^-8^
**Variables (validation set)**	**(n=31)**	**(n=32)**	
Average age,years	31.29 (5.72)	29.44 (4.84)	0.1711
Sexual abstinence time,days	4.03 (0.66)	3.88 (0.66)	0.3472
Semen parameters			
pH	7.34 (0.19)	7.44 (0.19)	0.0655
Total volume,mL	4.68 (1.02)	4.16 (1.70)	0.1038
Sperm parameters			
Sperm density,×10^6^/mL	49.13 (8.86)	44.11 (8.85)	0.0916
Sperm viability,%	67.98 (8.3)	15.46 (6.94)	7.14×10^-35^
Progressive motility (PR)^e^	50.2 (8.21)	10.88 (5.34)	3.34×10^-28^

a Data are presented as mean (SD).

b Normozoospermic men vs Asthenozoospermia patients.

e a,rapid progressive motility.

### Sample Preparation

Semen samples obtained through by masturbation after 3-5 days of abstinence and then were transferred into a 15 mL centrifuge tube (Corning) and liquefied for 30 min at 37°C. Sperm concentration and viability were assessed by Sperm analysis system (SAS medical). Routine semen analysis was based on the World Health Organization (WHO) criteria (22). The sperms isolated from semen samples by centrifuging at 3000 rpm for 5 min at room temperature were resuspended in PBS and stored at -80°C for further protein analysis.

### Isolation of Exosomes From Seminal Plasma

Differential centrifugation was employed to isolate exosomes from seminal plasma that was obtained by centrifuging semen samples (850 g, 5min at room temperature). In brief, cell debris was removed by spinning at low speed (3,000 g, for 30 min) at the first step. Then, the shedding vesicles and the other larger vesicles were removed by centrifugation at 10,000 g for 30 min. Finally, the exosome pellets were collected by centrifugation at 110,000 g for 70 min, and then re-suspended in PBS buffer, and the supernatant was kept as exosome-free seminal plasma. All procedures steps carried out at 4°C.

### Transmission Electron Microscopy Assay (TEM)

The morphology of exosome was imaged by TEM. Briefly, the exosome pellet was fixed in 2.5% glutaraldehyde overnight at 4°C, and then was rinsed with PBS and post-fixed with 1% osmium tetroxide for 1 h at room temperature. Then the exosome pellet was embedded in 10% gelatin and fixed with glutaraldehyde at 4°C and carved into blocks. Subsequently, the exosome pellet was dehydrated by incubation for 10 min with graded alcohol series (30%, 50%, 70%, 90%, 95%, and 100%, 3 times), then the samples were incubated with propylene oxide, and infiltrated with increasing concentrations of Quetol-812 epoxy resin mixed with propylene oxide (25%, 50%, 75%, and 100%). At last, exosome samples embedded in pure, fresh Quetol-812 epoxy resin and polymerized increasing temperature for 12-24 h (35°C for 12 h, 45°C for 12 h, and 60°C for 24 h), were cut into ultrathin sections using Leica UC6 ultramicrotome, and stained with uranyl acetate (10 min) and lead citrate (5 min) at room temperature. The samples were imaged with TEM (FEI Tecnai T20) at a voltage of 120 kV.

### Illumina High-Throughput Sequencing

Total RNA from pooled sperm samples of normozoospermic men and asthenozoospermia patients (each pooled from 10 individuals) was prepared using TRIzol Reagent (Takara, Dalian, China). About 1~2 μg of quantified total RNA was performed high-throughput sequencing on Illumina NextSeq500 system according to the manufacturer’s instructions. The sequences corresponding to known piRNAs after data analysis were determined by perfect sequence matching to the piRNA database piRNABank (http://pirnabank.ibab.ac.in/). All data have been uploaded to the GEO database (Accession number: GSE172486).

### RNA Isolation and qRT-PCR Assays

TRIzol Reagent (TaKaRa, Dalian, China) was used to isolate total RNA from sperm, exosome and exosome-free seminal plasma. Briefly, sperms derived from 1 ml of semen, exosome from 100 μl of seminal plasma and 100 μl of exosome-free seminal plasma were mixed with 1 ml TRIzol, after being vortexed vigorously for 10 s, the mixture was incubated with 200 μl of chloroform for 10 min on ice, The RNA containing phase was then transferred to a fresh RNase-free tube after centrifugation at 16,000 g for 20 min at 4°C, Then the supernatant was incubated with equal volume of isopropanol at -20°C for 1.5 h to precipitate RNA. The isolated RNA was collected by centrifugation (16,000 g, 4°C, 20 min), was then washed rinsed once with 75% ethanol and dried for 20 min at room temperature. Finally, the RNA was dissolved in 20 μl RNase-free H_2_O and stored at -80°C for further analysis.

Total RNA (2 μl) was reverse transcribed to cDNA using AMV reverse transcriptase based on the manufacturer’s instruction. Then, 1 μl of cDNA was used for subsequent qRT-PCR analysis on a Roche LightCycler 480 PCR system. Taqman piRNA probes (GenePharma, Shanghai, China) was used to measure the piRNA level in this study. All reactions were performed in triplicate. RNU6-6P was used as an internal control.

### Protein Extraction and Western Blotting

Sperms were lysed in RIPA lysis buffer with freshly added PMSF for 30 min on ice, and sonication (Sonics&materials Inc.VCX 130 PB) was used to facilitate sperm cell disruption, Insoluble debris were removed by centrifugation at 16,000 g, 4°C for 10 min. The protein concentration was quantified using a BCA proteinassay kit (Thermo Scientific, Rockford, IL, USA). The expression of indicated proteins were detected using their specific antibodies, including anti-MitoPLD antibody (ab170183) and anti-PIWIL1 (ab12337) purchased from Abcam (Cambridge, MA, USA), β-actin antibody (sc-69879) was served as the reference, which purchased from Santa Cruz (Dallas, TX, USA). Western blot image acquisition was performed using the Bio-Rad ChemiDoc imaging system, and Image J was used for densitometric analysis.

### Statistical Analysis

All images are representative of at least three different experiments. The data shown are the mean ± SE of at least three independent experiments. Student’s t-test was used for statistical analysis and *p* value < 0.05 (indicated by *), < 0.01 (indicated by **) or < 0.001 (indicated by ***) were considered statistically significant.

## Results

### Characterization of Exosomes From Seminal Plasma

**Figure 1:**
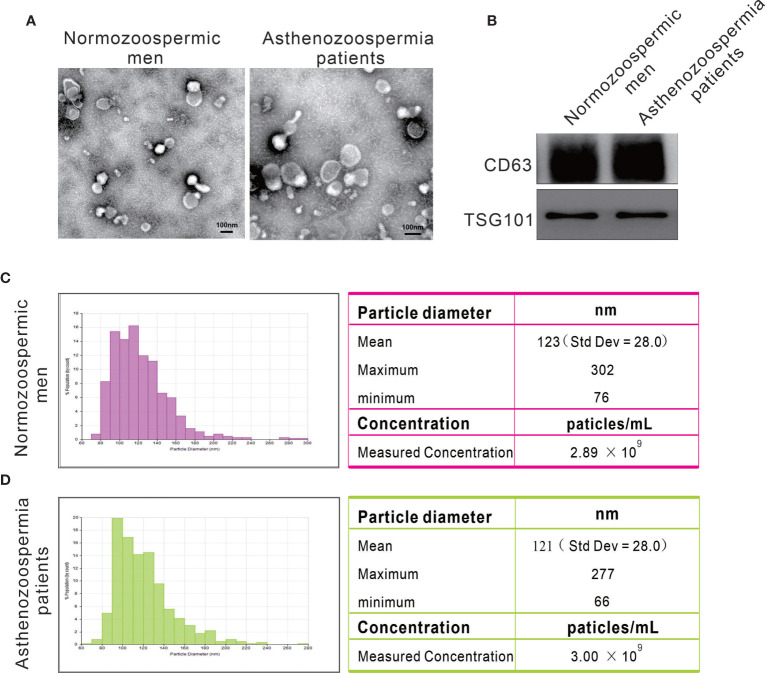
Particles isolated from seminal plasma exhibit the characteristic features of exosomes. **(A)** Representative transmission electron micrographs of exosomes. **(B)** Western blots on exosomes using antibodies against the common exosomal markers CD63 and TSG101. **(C, D)** Representative particle size distribution profiles from seminal plasma of normozoospermic men and asthenozoospermia patients.

We purified exosomes from seminal plasma of normozoospermic men and asthenozoospermia patients by ultracentrifugation and examined the particle size and morphology by transmission electron microscopy. Under electron microscopy, the exosomes isolated from both normozoospermic men and asthenozoospermia patients appeared as lipid bilayer-bound single and small clumps of particles of 30-150 nm in diameter, consistent with the expected size range of exosomes (**Figure 1A**). The exosomes isolated from normozoospermic men and asthenozoospermia patients were further identified by the presence of equal amounts of universal exosomal markers (CD63 and TSG101) based on immunoblotting (**Figure 1B**). Moreover, the particle size and concentration of exosomes were determined by nanoparticle tracking analysis. Exosomes from normozoospermic men had an average diameter of 123 nm, most exosomes (> 85%) range from 80 to 150 nm, and the concentration of exosomes was 2.89×10^9^ particles/mL (**Figure 1C**). For exosomes from asthenozoospermia patients, the mean diameter was 121 nm, and the concentration was 3.0×10^9^ particles/mL (**Figure 1D**). The results confirmed that exosomes were present in high concentrations in human seminal plasma, but the size range and concentration of exosomes were not differentially present between normozoospermic men and asthenozoospermia patients.

### Differentially Expressed piRNAs in Seminal Plasma Exosomes

**Figure 2:**
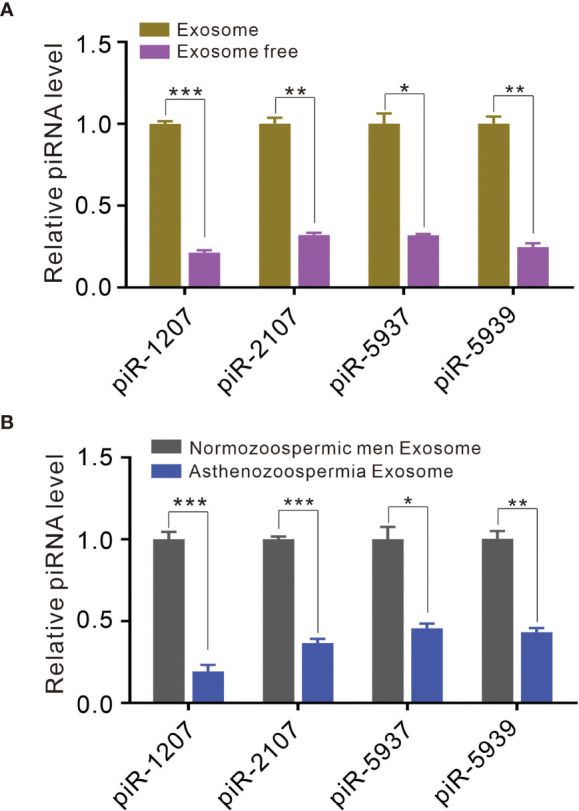
Exosomes contain piRNAs and differential presence of piRNAs between asthenozoospermia patients and normozoospermic men. **(A)** Quantitative RT-PCR analysis of *piR-1207, piR-2107, piR-5937 and piR-5939 in* exosomes and exosome-free seminal plasma. **(B)** Quantitative RT-PCR analysis of *piR-1207*, *piR-2107*, *piR-5937* and *piR-5939* in exosomes from asthenozoospermia patients and normozoospermic men. *p < 0.05; **p < 0.01; ***p < 0.001

A previous study has identified a great amount of small RNA in the exosomal fraction of seminal plasma (21). In this study, we determined to compared the ratio of piRNA levels in exosomes to that in exosome-free seminal plasma. We selected *piR-1207*, *piR-2107*, *piR-5937* and *piR-5939* as the representative piRNAs and measured their levels in exosomes and exosome-free seminal plasma by quantitative RT-PCR assay because our previous study have identified these piRNAs as the significantly downregulated piRNAs in seminal plasma of asthenozoospermia patients compared with normozoospermic men (3). *piR-1207*, *piR-2107*, *piR-5937* and *piR-5939* were mainly stored in exosomes (**Figure 2A**), suggesting that the majority of piRNAs were present in exosomal fraction of seminal plasma. Meanwhile, we also compared the levels of these piRNAs in exosomes between normozoospermic men and asthenozoospermia patients. The result proved that *piR-1207*, *piR-2107*, *piR-5937* and *piR-5939* were significantly reduced in exosomes from asthenozoospermia patients compare with the normozoospermic men (**Figure 2B**). These results indicated that the reduction of piRNAs in exosomes contributed to the reduction of piRNAs in seminal plasma of asthenozoospermia patients.

### Profiling Sperm piRNAs by High-Throughput Sequencing

**Figure 3:**
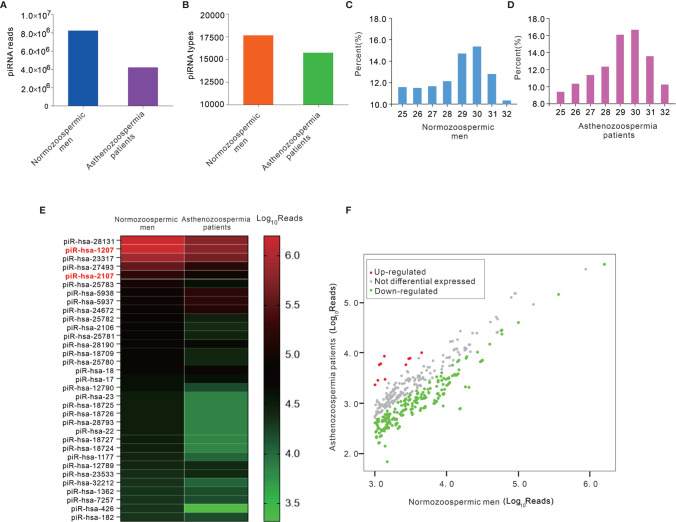
The analysis of high-throughput sequencing of piRNAs in sperms from asthenozoospermia patients and normozoospermic men. **(A, B)** Total sequencing reads **(A)** and types **(B)** of piRNAs were decreased in sperms of asthenozoospermia patients compared with those in normozoospermic men. **(C, D)** The distribution of small RNAs of various lengths (25-32 bp) measured by high-throughput sequencing in sperms. **(E, F)** The heatmap **(E)** and scatter plots **(F)** analysis of sequencing reads of piRNAs between asthenozoospermia patients and normozoospermic men.

Although piRNAs were significantly reduced in exosomes of asthenozoospermia patients, the source and mechanism for the dysregulation of piRNAs remain obscure. piRNAs are expressed abundantly in pachytene spermatocytes and round spermatids (23). Given that exosomes are likely secreted from multiple cellular sources in the male genital tract, we speculate that piRNAs may be actively secreted from spermatocytes during the spermatogenesis process. However, it is very difficult to get spermatocytes from asthenozoospermia patients. Alternatively, we systematically characterized the piRNA profiles in mature sperms. Total RNA was extracted from pooled sperm samples of normozoospermic men and asthenozoospermia patients (each pooled from 10 individuals), and was qualified by agarose gel electrophoresis and quantified using Nanodrop. Subsequently, commercial kit was used for piRNA-seq library preparation, which includes 3’-adapter and 5’-adapter ligation, cDNA synthesis and library PCR amplification. The prepared piRNA-seq libraries were finally quantified using Agilent BioAnalyzer 2100, then sequenced by using Illumina NextSeq 500. After removing 5’and 3’ adaptor sequences and aligned to the piRBase, a total of 8,245,354 and 4,220,714 reads of piRNAs were obtained in the sperm of normozoospermic men group and asthenozoospermia patient group, respectively (**Figure 3A**). Moreover, the types of piRNAs were decreased from sperm of normozoospermic men group (17657) to asthenozoospermia patient group (15742) (**Figure 3B**). Analysis of the length distribution revealed that sperm of normozoospermic men group and asthenozoospermia patient group contained a number of small RNAs with size that was consistent with common the size of piRNAs (25–32 nucleotides) (**Figures 3C, D**). Next, we narrowed the list of piRNAs. Firstly, we narrowed down and selected 33 known highly expressed piRNAs under the condition of sequencing reads are larger than 20000 in the group of normozoospermic men, Heatmap analysis was performed based on these criteria, the result showed that 17 piRNAs have at least 2-fold higher reads in normozoospermic men group than in the asthenozoospermia patient group (**Figure 3E**, **Supplementary Table 1**). Subsequently, we compared asthenozoospermia patient group with the normozoospermic men group by scatter plots with the following parameters: sequencing reads are larger than 1000 in the group of normozoospermic men. The result was shown in **Figure 3F** and **Supplementary Table 2**, indicating a remarkable different expression level of piRNAs between asthenozoospermia patient group and normozoospermic men group. Generally, the comparison among the sperm piRNA profiles revealed a considerable reduction of sperm piRNAs in asthenozoospermia patients relative to normozoospermic men (from 8,245,354 to 4,220,714 reads and from 17,657 to 15,742 types of piRNAs).

### Individual Quantification of Sperm piRNAs by Quantitative RT-PCR

**Figure 4:**
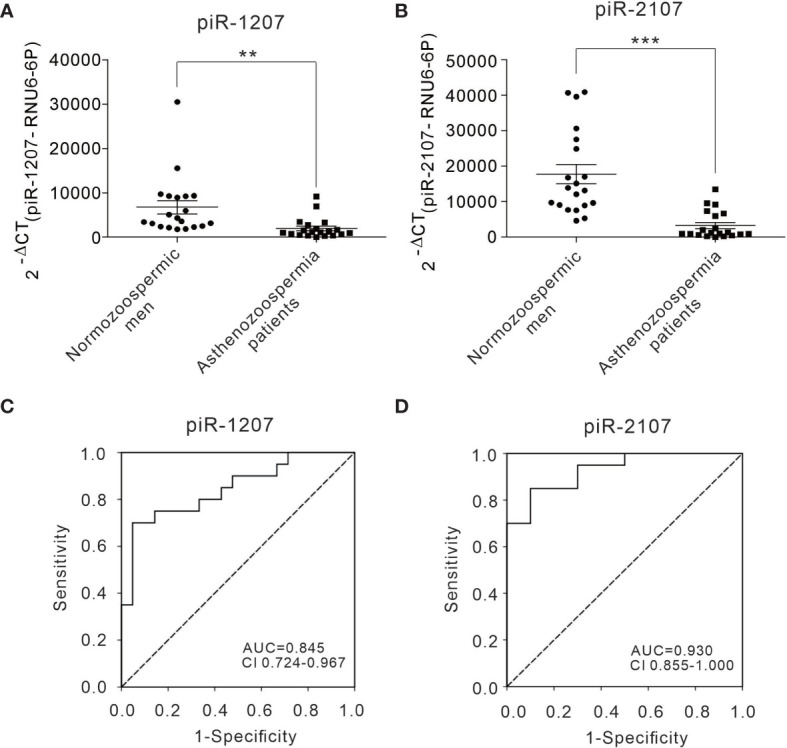
Differential presence of piRNAs from sperms between asthenozoospermia patients and normozoospermic men and ROC curve analysis. **(A, B)** The relative level of *piR-1207* and *piR-2107* were determined by quantitative RT-PCR in the sperms of asthenozoospermia patients (n=20) compared with those in the sperms of normozoospermic men (n=20). **(C, D)** ROC curve for the *piR-1207* **(C)** and *piR-2107* **(D)** to separate asthenozoospermia patients from normozoospermic men. **p < 0.01; ***p < 0.001

Next, a TaqMan probe-based quantitative RT-PCR assay was performed to measure the presence of piRNAs in individual samples. The representative *piR-1207* and *piR-2107* were assessed in sperm samples of 20 normozoospermic men and 20 asthenozoospermia patients. Consistent with the results from deep sequencing, *piR-1207* and *piR-2107* levels were markedly downregulated in sperm of asthenozoospermia patients (**Figures 4A, B**). We further performed receiver-operating characteristic (ROC) curve analysis to assess the usefulness of *piR-1207* and *piR-2107* in discriminating asthenozoospermia patients from normozoospermic men. ROC curve analysis for *piR-1207* revealed an AUC of 0.845 and an optimal cut-off value of 1717.93 with a corresponding sensitivity of 70.0% and specificity of 95.2% (**Figure 4C**), and for *piR-2107* revealed an AUC of 0.93 and an optimal cut-off value of 7476.52 with a corresponding sensitivity of 85% and specificity of 90% (**Figure 4D**). These results showed that *piR-1207* and *piR-2107* could serve as valuable indicators for distinguishing asthenozoospermia patients from normozoospermic men.

### Expression Level of MitoPLD Is Decreased in Asthenozoospermia Patient Sperms

**Figure 5:**
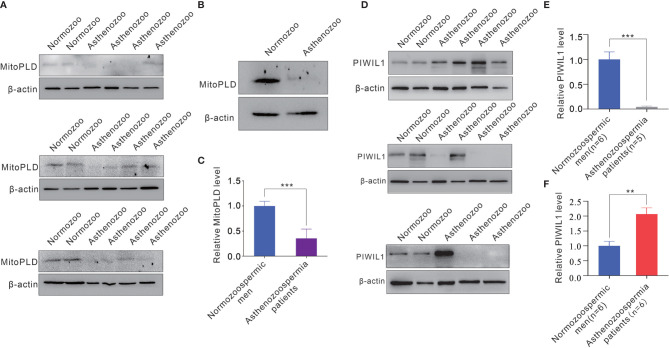
The expression level of MitoPLD and PIWIL1 protein from sperms between asthenozoospermia patients and normozoospermic men. **(A)** Western blotting analysis of the expression levels of MitoPLD in individual sperm samples between asthenozoospermia patients and normozoospermic men. **(B)** Western blotting analysis of the expression levels of MitoPLD in pooled sperm samples between asthenozoospermia patients and normozoospermic men (each pooled from 10 individuals). **(C)** Quantitative analysis of panel **(A, B)**. **(D)** Western blotting analysis of the expression levels of PIWIL1 in individual sperm samples between asthenozoospermia patients and normozoospermic men. **(E, F)** Quantitative analysis of expression levels of PIWIL1 were decreased **(E)** or increased **(F)** in sperms of asthenozoospermia patients. **p < 0.01; ***p < 0.001.

Since the biogenesis and function of piRNA is tightly associated with that of the PIWI protein subfamily, and MitoPLD is essential for the creation of the 5’ ends of primary piRNAs, the marked reduction of mature piRNAs in sperm of asthenozoospermia patients suggested the dysfunction or loss of expression of these essential enzymes in sperms. Therefore, we measured the expression level of MitoPLD and PIWIL1 in the sperms of normozoospermic men and asthenozoospermia patients. The results revealed that the expression of MitoPLD protein was significantly reduced in asthenozoospermia patient sperms (**Figures 5A–C**). In contrast, the alteration of PIWIL1 in asthenozoospermia patient sperms was irregular, PIWIL1 expression level were increased in the sperms of some asthenozoospermia patients (6 out of 11 patients), but were significantly decreased in 5 patients (**Figures 5D–F**).

## Discussion

Although piRNAs have been found to have obvious expression in germ cells across various animal species, specifically in male germ cells of mammals, researches related to their detailed function and mechanism remain obscure. Up to now, what is becoming clear is that piRNAs are participate in posttranscriptional regulation of protein-coding genes as well as in the repression of retrotransposons and are indispensable for male fertility, and a recent study showed that PIWI/piRNA pathway genes repression by hypermethylation is probably contributed to unsuccessful spermatogenesis (24). These results suggest that the critical role and function of piRNAs during spermatogenesis process has been well documented, whether piRNAs in sperm may regulate sperm motility remain largely unknown. Increasing studies have shown that miRNAs expressed in mature sperm could regulate sperm motility (25, 26). In contrast to miRNAs, piRNAs were just discovered in 2006, and the biogenesis and functions remain largely unexplored. However, piRNAs are known to be much more abundant and germ cell-specific than miRNAs, suggesting that piRNAs may potentially play a more fundamental role in regulating the sperm motility. In this study, we found that piRNAs were enriched in sperms and observed that a massive amount of piRNAs were lost in sperms of asthenozoospermia patients. Furthermore, ROC curve analysis revealed a strong relationship between the low presence of sperm piRNAs and asthenozoospermia patients, suggesting that sperm piRNAs may be essential for sperm motility. The biological roles of these piRNAs in sperm motility with male infertility, which may provide some pathophysiological clues for the molecular mechanisms of this disease, call for further investigations.

Based on their biogenesis models, they are typically classified into two groups: the primary processing pathway and amplification loop (secondary processing pathway). MitoPLD, a mitochondria-anchored endonuclease belonging to the member of the phospholipase D superfamily proteins, is conserved among diverse species and is implicated in the primary processing pathway of piRNAs. MitoPLD is previously known to be a phospholipase that hydrolyzes cardiolipin to generate phosphatidic acid and is involved in the regulation of mitochondrial morphology (27, 28). MitoPLD is also implicated in the formation of nuage (also known as inter-mitochondrial cement or chromatoid body), which works as a pivotal cytoplasmic structure comprising most piRNA-related proteins (15, 27). Surprisingly, MitoPLD was found to be essential to piRNA biogenesis. MitoPLD acts as an endonuclease and conducts the first cleavage of piRNAs precursors to generate the 5’ ends of secondary piRNAs, and then the cleaved piRNAs are transferred to PIWI proteins to trigger the secondary piRNA processing pathway (29, 30). Knockout of MitoPLD abolishes the majority of piRNA in male germ cells and resulted in transposon activation and arrest of spermatogenesis, characteristic phenotypes of piRNA pathway mutants (12).In this study, we measured the expression levels of MitoPLD in sperms of normozoospermic men and asthenozoospermia patients and found that MitoPLD protein was significantly downregulated in sperms of asthenozoospermia patients. Massive reduction of piRNAs in the sperms of asthenozoospermia patients may be caused, at least in part, by the parallel reduction of MitoPLD protein in sperms. Further studies are required to investigate whether dysregulation or dysfunction of MitoPLD is involved in the pathology of infertility.

Exosomes are nano-sized vesicles with a diameter ranging between 30 and 150 nm. Released by multiple cell types, exosomes are present in a variety of body fluids and can transfer bioactive molecules (e.g., proteins, lipids and nucleic acids) between neighboring and distant cells (31). Scientists have reached a consensus that exosomes play a key role in intercellular communication *via* the horizontal transfer of miRNAs. However, the presence of piRNAs in exosomes has only currently been noted. A recent study has shown that a large number of small RNAs (including miRNAs and piRNAs) were contained and protected within seminal exosomes (21). In this study, we also identified a majority of piRNAs in the seminal exosomes. In addition, we found that piRNAs in seminal exosomes of asthenozoospermia patients were significantly decreased compared with normozoospermic men. However, it has not been established yet if the piRNA in seminal exosomes can have regulatory functions in the recipient cells and act as a new role in the intercellular communication system. Future studies are needed to characterize the functions of piRNAs in seminal exosomes and to investigate the role of communicators of exosomal piRNAs in the microenvironment of genital tract.

In conclusion, we systematically characterized the piRNA profiles in sperms of normozoospermic men and asthenozoospermia patients and found that the amounts of piRNAs were significantly decreased in the sperms of asthenozoospermia patients. We also investigated the mechanism for the dysregulation of piRNAs in sperms and revealed that the parallel reduction of MitoPLD may be the cause and consequence of male infertility.

## Data Availability Statement

The datasets presented in this study can be found in online repositories. The names of the repository/repositories and accession number(s) can be found below: NCBI GEO repository, GSE172486.

## Ethics Statement

The studies involving human participants were reviewed and approved by Hangzhou Medical College Ethics Committee, Nanjing Drum Tower Hospital Ethics Committee. The patients/participants provided their written informed consent to participate in this study.

## Author Contributions

Study conceptualization: YH and JZ. Data acquisition, analysis and interpretation: YH, YW, CY, and LS. Clinical samples and data collection: XZ. Technical or material support: LC, HC, and FG. Manuscript writing and editing: YH, CY, LS, and JZ. All authors contributed to the article and approved the submitted version.

## Funding

This study was supported by a grant from the National Natural Science Foundation of China (No. 81801513).

## Conflict of Interest

The authors declare that the research was conducted in the absence of any commercial or financial relationships that could be construed as a potential conflict of interest.

## References

[1] Abu-Halima M Ludwig N Hart M Leidinger P Backes C Keller A. Altered Micro-Ribonucleic Acid Expression Profiles of Extracellular Microvesicles in the Seminal Plasma of Patients With Oligoasthenozoospermia. Fertil Steril (2016) 106(5):1061–9.e3. 10.1016/j.fertnstert.2016.06.030 27424049

[2] Cardona Barberan A Boel A Vanden Meerschaut F Stoop D Heindryckx B. Diagnosis and Treatment of Male Infertility-Related Fertilization Failure. J Clin Med (2020) 9(12):3899. 10.3390/jcm9123899 PMC7761017 33271815

[3] Hong Y Wang C Fu Z Liang H Zhang S Lu M. Systematic Characterization of Seminal Plasma Pirnas as Molecular Biomarkers for Male Infertility. Sci Rep (2016) 6:24229. 10.1038/srep24229 27068805 PMC4828650

[4] Wang C Yang C Chen X Yao B Yang C Zhu C. Altered Profile of Seminal Plasma Micrornas in the Molecular Diagnosis of Male Infertility. Clin Chem (2011) 57(12):1722–31. 10.1373/clinchem.2011.169714 21933900

[5] Okada H Tajima A Shichiri K Tanaka A Tanaka K Inoue I. Genome-Wide Expression of Azoospermia Testes Demonstrates a Specific Profile and Implicates ART3 in Genetic Susceptibility. PLoS Genet (2008) 4(2):e26. 10.1371/journal.pgen.0040026 18266473 PMC2233677

[6] Lau NC Seto AG Kim J Kuramochi-Miyagawa S Nakano T Bartel DP. Characterization of the Pirna Complex From Rat Testes. Science (2006) 313(5785):363–7. 10.1126/science.1130164 16778019

[7] Grivna ST Beyret E Wang Z Lin H. A Novel Class of Small Rnas in Mouse Spermatogenic Cells. Genes Dev (2006) 20(13):1709–14. 10.1101/gad.1434406 PMC1522066 16766680

[8] Aravin A Gaidatzis D Pfeffer S Lagos-Quintana M Landgraf P Iovino N. A Novel Class of Small Rnas Bind to MILI Protein in Mouse Testes. Nature (2006) 442(7099):203–7. 10.1038/nature04916 16751777

[9] Girard A Sachidanandam R Hannon GJ Carmell MA. A Germline-Specific Class of Small Rnas Binds Mammalian Piwi Proteins. Nature (2006) 442(7099):199–202. 10.1038/nature04917 16751776

[10] Aravin AA Hannon GJ Brennecke J. The Piwi-Pirna Pathway Provides an Adaptive Defense in the Transposon Arms Race. Science (2007) 318(5851):761–4. 10.1126/science.1146484 17975059

[11] Kim VN Han J Siomi MC. Biogenesis of Small Rnas in Animals. Nat Rev Mol Cell Biol (2009) 10(2):126–39. 10.1038/nrm2632 19165215

[12] Watanabe T Chuma S Yamamoto Y Kuramochi-Miyagawa S Totoki Y Toyoda A. MITOPLD is a Mitochondrial Protein Essential for Nuage Formation and Pirna Biogenesis in the Mouse Germline. Dev Cell (2011) 20(3):364–75. 10.1016/j.devcel.2011.01.005 PMC3062204 21397847

[13] Nishimasu H Ishizu H Saito K Fukuhara S Kamatani MK Bonnefond L. Structure and Function of Zucchini Endoribonuclease in Pirna Biogenesis. Nature (2012) 491(7423):284–7. 10.1038/nature11509 23064230

[14] Voigt F Reuter M Kasaruho A Schulz EC Pillai RS Barabas O. Crystal Structure of the Primary Pirna Biogenesis Factor Zucchini Reveals Similarity to the Bacterial PLD Endonuclease Nuc. RNA (2012) 18(12):2128–34. 10.1261/rna.034967.112 PMC3504665 23086923

[15] Kabayama Y Toh H Katanaya A Sakurai T Chuma S Kuramochi-Miyagawa S. Roles of MIWI, MILI and PLD6 in Small RNA Regulation in Mouse Growing Oocytes. Nucleic Acids Res (2017) 45(9):5387–98. 10.1093/nar/gkx027 PMC5435931 28115634

[16] Brennecke J Aravin AA Stark A Dus M Kellis M Sachidanandam R. Discrete Small RNA-Generating Loci as Master Regulators of Transposon Activity in Drosophila. Cell (2007) 128(6):1089–103. 10.1016/j.cell.2007.01.043 17346786

[17] Gunawardane LS Saito K Nishida KM Miyoshi K Kawamura Y Nagami T. A Slicer-Mediated Mechanism for Repeat-Associated Sirna 5’ End Formation in Drosophila. Science (2007) 315(5818):1587–90. 10.1126/science.1140494 17322028

[18] Gou LT Dai P Yang JH Xue Y Hu YP Zhou Y. Pachytene Pirnas Instruct Massive Mrna Elimination During Late Spermiogenesis. Cell Res (2014) 24(6):680–700. 10.1038/cr.2014.41 24787618 PMC4042167

[19] Zhang P Kang JY Gou LT Wang J Xue Y Skogerboe G. MIWI and Pirna-Mediated Cleavage of Messenger Rnas in Mouse Testes. Cell Res (2015) 25(2):193–207. 10.1038/cr.2015.4 25582079 PMC4650574

[20] Dai P Wang X Gou LT Li ZT Wen Z Chen ZG. A Translation-Activating Function of MIWI/Pirna During Mouse Spermiogenesis. Cell (2019) 179(7):1566–81.e16. 10.1016/j.cell.2019.11.022 31835033 PMC8139323

[21] Vojtech L Woo S Hughes S Levy C Ballweber L Sauteraud RP. Exosomes in Human Semen Carry a Distinctive Repertoire of Small Non-Coding Rnas With Potential Regulatory Functions. Nucleic Acids Res (2014) 42(11):7290–304. 10.1093/nar/gku347 PMC4066774 24838567

[22] World Health Organization. WHO Laboratory Manual for the Examination and Processing of Human Semen. 5th ed Vol. xiv. Geneva: World Health Organization (2010). 271 p.p.

[23] He Z Kokkinaki M Pant D Gallicano GI Dym M. Small RNA Molecules in the Regulation of Spermatogenesis. Reproduction (2009) 137(6):901–11. 10.1530/REP-08-0494 19318589

[24] Zhang GW Wang L Chen H Guan J Wu Y Zhao J. Promoter Hypermethylation of PIWI/Pirna Pathway Genes Associated With Diminished Pachytene Pirna Production in Bovine Hybrid Male Sterility. Epigenetics (2020) 15(9):914–31. 10.1080/15592294.2020.1738026 PMC7518677 32141383

[25] Curry E Safranski TJ Pratt SL. Differential Expression of Porcine Sperm Micrornas and Their Association With Sperm Morphology and Motility. Theriogenology (2011) 76(8):1532–9. 10.1016/j.theriogenology.2011.06.025 21872314

[26] Wang W Liang K Chang Y Ran M Zhang Y Ali MA. Mir-26a is Involved in Glycometabolism and Affects Boar Sperm Viability by Targeting PDHX. Cells (2020) 9(1):146. 10.3390/cells9010146 PMC7016825 31936222

[27] Huang H Gao Q Peng X Choi SY Sarma K Ren H. Pirna-Associated Germline Nuage Formation and Spermatogenesis Require Mitopld Profusogenic Mitochondrial-Surface Lipid Signaling. Dev Cell (2011) 20(3):376–87. 10.1016/j.devcel.2011.01.004 PMC3061402 21397848

[28] Choi SY Huang P Jenkins GM Chan DC Schiller J Frohman MA. A Common Lipid Links Mfn-Mediated Mitochondrial Fusion and SNARE-Regulated Exocytosis. Nat Cell Biol (2006) 8(11):1255–62. 10.1038/ncb1487 17028579

[29] Brennecke J Malone CD Aravin AA Sachidanandam R Stark A Hannon GJ. An Epigenetic Role for Maternally Inherited Pirnas in Transposon Silencing. Science (2008) 322(5906):1387–92. 10.1126/science.1165171 PMC2805124 19039138

[30] Ishizu H Siomi H Siomi MC. Biology of PIWI-Interacting Rnas: New Insights Into Biogenesis and Function Inside and Outside of Germlines. Genes Dev (2012) 26(21):2361–73. 10.1101/gad.203786.112 PMC3489994 23124062

[31] Zhou Y Zhou G Tian C Jiang W Jin L Zhang C. Exosome-Mediated Small RNA Delivery for Gene Therapy. Wiley Interdiscip Rev RNA (2016) 7(6):758–71. 10.1002/wrna.1363 27196002

